# The Impact of Relaxation Massage Prior to Bedtime on Sleep Quality and Quantity in People with Symptoms of Chronic Insomnia: A Home-Based Sleep Study

**DOI:** 10.3390/healthcare13020180

**Published:** 2025-01-17

**Authors:** Ilias Ntoumas, Christina Karatzaferi, Christoforos D. Giannaki, Fotini Papanikolaou, Aggelos Pappas, Efthimios Dardiotis, Giorgos K. Sakkas

**Affiliations:** 1 Department of Physical Education and Sport Science, School of PE, Sports and Dietetics, University of Thessaly, 42100 Trikala, Greece; intoumas@uth.gr (I.N.); ck@uth.gr (C.K.); papanikolf@gmail.com (F.P.); apappas@pe.uth.gr (A.P.); 2 Department of Life Sciences, School of Life and Health Sciences, University of Nicosia, 2417 Nicosia, Cyprus; giannaki.c@unic.ac.cy; 3 Research Centre for Exercise and Nutrition (RECEN), 2417 Nicosia, Cyprus; 4 Department of Neurology, School of Medicine, University of Thessaly, 41110 Larisa, Greece; edar@med.uth.gr

**Keywords:** sleep, well-being, health, EEG, efficiency, sleepiness, lifestyle

## Abstract

**Background/Objectives:** Manual massage is an effective treatment approach for reducing general stress and promoting an overall sense of well-being. Relaxation massage aims to alleviate psychophysiological tension, enhance both blood and lymphatic circulation, and promote mental and physical relaxation. It is particularly beneficial for those with anxiety-related symptoms (such as generalized anxiety disorder and social anxiety) and sleep disorders, aiming to improve calmness and promote sleepiness. **Aims:** The purpose of the present study was to assess the effectiveness of a single session of relaxation massage prior to bedtime on sleep quality and quantity indices in individuals with symptoms of chronic insomnia. **Methods**: In total, 20 (N = 20) healthy individuals (aged 25.5 ± 12.0 years; 6F/14M) with a score on the Athens Insomnia Scale of ≥16 participated under three different conditions over one week apart: (1) a 45 min relaxation massage condition (REL), (2) a 45 min sham massage condition (PLA), and (3) a control condition with no massage. Sleep activity was monitored using a portable polysomnographic system. **Results**: A statistically significant effect was observed between sleep efficiency across the three sessions (*p* = 0.034), with a notable effect in the relaxation massage (REL) session (*p* = 0.045). Additionally, sustained sleep efficiency showed a statistically significant difference among the sessions (*p* = 0.005). **Conclusions**: Relaxation massage prior to bedtime could be used as an effective and safe non-pharmacological approach for improving sleep efficiency and potentially restoring the fragmented sleep of individuals with symptoms of insomnia. Trial registration number: The trial was registered at clinicaltrials.gov as NCT06781866.

## 1. Introduction

Sleep is a naturally occurring state for both the mind and body, marked by changes in consciousness, decreased sensory responsiveness, lowered muscle activity, and the suppression of almost all voluntary muscles [1]. There is no fixed amount of hours of sleep per night; however, it is recommended that for a healthy adult, the minimum hours of uninterrupted sleep should be approximately 6–8 h [2]. In modern Western societies, sleep deprivation seems to have become a new epidemic [3]. Indeed, sleep disorders have greatly increased in all age groups, and they are related mostly to an unhealthy lifestyle [4]. In particular, insomnia is one of the most common sleep disorders that affects the health and quality of life of the sufferer [5]. Recent global statistics show that sleep disorders affect almost 10% of the general population, with chronic insomnia reaching 33% of those [6]. Insomnia has been associated with reduced cardiorespiratory fitness (CRF), which is a significant risk factor for cardiovascular diseases [7]. Specifically, the study by Zou et al. demonstrated a significant association between insomnia and lower CRF, suggesting that insomnia may affect cardiometabolic health through physical fitness [8]. On the contrary, poor physical fitness may also exacerbate insomnia, creating a bidirectional relationship between these conditions [9]. Furthermore, poor sleep quality, associated with insomnia, has been linked to an increased risk of developing chronic conditions associated with modern lifestyles, including diabetes, hypertension, dyslipidemia, metabolic syndromes, and cardiovascular diseases [10,11]. Studies suggest that insomnia negatively impacts health through mechanisms such as elevated cortisol levels, reduced immunity, and increased sympathetic activity, all of which contribute to the development of chronic non-communicable diseases [12].

The most common treatment of insomnia is pharmacological; however, these drugs come with significant side effects, can become addictive with prolonged use, and do not help to restore sleep quality [13]. Cognitive behavioral therapy for insomnia (CBTi) is the first-line treatment for insomnia. Additionally, acceptance and commitment therapy (ACT) has proven effective in improving sleep quality and emotional regulation. Specifically, Zakiei et al. showed that ACT significantly enhanced sleep quality and emotional processing in individuals with insomnia, with benefits lasting for up to 12 weeks [14].

Lifestyle medicine is a branch of medicine that aims to maintain optimal health and prevent, treat, and reverse chronic diseases via lifestyle changes [15]. This approach is based on eight pillars, one of which is improving sleep hygiene [16]. Lifestyle medicine proposes a series of interventions to improve sleep quality [17], and one of those is to promote relaxation and calmness before bedtime. In this direction, we explored the impact of full-body relaxation massage prior to bedtime with the aim to promote relaxation and help people with insomnia symptoms improve their sleep quality and quantity. Massage therapy is a healing practice that dates back thousands of years [18], and it has been used as a tool to reduce stress levels, improve blood circulation and muscle relaxation, and promote an overall sense of calmness [19].

The application of massage has been shown to reduce stress and fatigue [20], improve sleep quality [21], reduce the severity of sleep disorders [22], reduce pain [23], and contribute to overall physical and mental well-being [24]. The technique of relaxation massage focuses on promoting psychosomatic relaxation through gentle, rhythmic movements and deep, light pressure [25]. It employs long, continuous strokes aimed at reducing stress and inducing a deep sense of relaxation, without targeting intense muscle pressure or enhancing blood circulation, as seen in other forms of massage, such as Swedish or sports massage [26]. The gentle nature of the technique allows for the activation of the parasympathetic nervous system, contributing to a decrease in heart rate, relaxation of the nervous system, and improvement in mental health [27].

Therefore, the primary aim of the current study was to assess the effect of a single session of relaxation massage prior to bedtime on sleep quality and quantity indices in individuals suffering from symptoms of insomnia. By investigating these parameters, we sought to determine whether relaxation massage can serve as an effective non-pharmacological intervention for improving sleep patterns and reducing sleep disturbances in individuals with chronic insomnia symptoms. The findings will contribute to the growing body of evidence regarding the potential therapeutic benefits of massage therapy for sleep disorders and overall well-being.

## 2. Materials and Methods

### 2.1. Subjects

**Figure 1:**
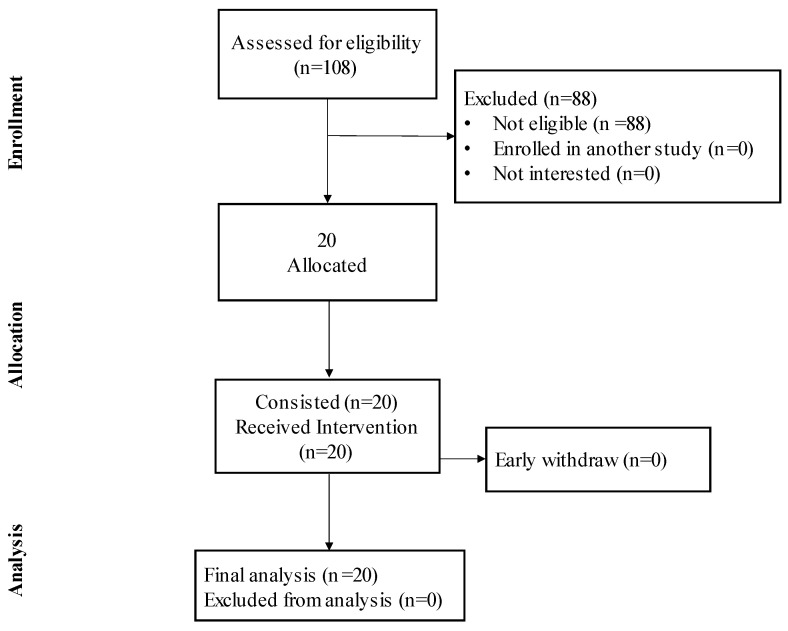
Consort diagram.

Twenty (N = 20) healthy participants (aged 25.5 ± 12.0 years; 6F/14M) fulfilled the inclusion criteria and participated in this study (Figure 1). The inclusion criteria included subjects of both sexes, aged >18 years, and with scores of ≥16 on the Athens Insomnia Scale (AIS-8). The exclusion criteria included a history of mental illness, dermatological diseases, allergies to massage oil, and any acute or chronic condition that would limit the ability of the patient to participate or consent to this study.

### 2.2. Study Design

**Figure 2:**
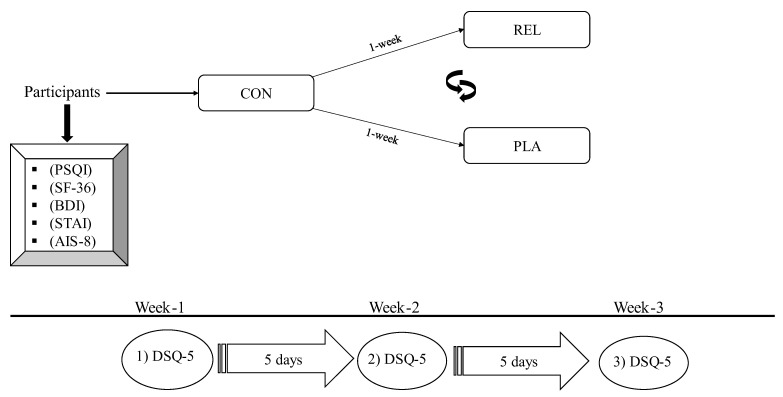
Study flow chart. Subjects were assigned in random orders to each of the two massage sessions. Note: (CON) control protocol; (REL) relaxation massage protocol; (PLA) placebo/sham massage protocol; (PSQI) Pittsburgh Sleep Quality Index; (BDI) Beck’s Depression Inventory; (STAI) State–Trait Anxiety Inventory; (DSQ-5) Daily Sleep Questionnaire–5 Days; (AIS-8) Athens Insomnia Scale. The order of either the REL or PLA allocation was random. The CON was always performed first as a baseline polysomnographic assessment.

The subjects participated in 3 different sessions, each 7 days apart, for 3 continuous weeks. In particular, “Session 1” was the control (CON) session, where participants did not receive any massage session and followed their usual bedtime routine. For “Session 2”, participants received a relaxation massage (REL) session lasting 45 min prior to bedtime, followed by their usual bedtime routine. For “Session 3”, participants received a “sham” massage (PLA) session lasting 45 min prior to bedtime, followed by their usual bedtime routine. While Session 1 was always performed first, Sessions 2 and 3 were randomly assigned to each participant (Figure 2). Both massage sessions were delivered by a professional masseur, while the researcher analyzing the data was blind to the assigned session. Sleep quality and quantity were assessed in all three sessions. This study was approved by the Human Research and Ethics Committee of the University of Thessaly (2334-3/4-2/7.2.2024). All subjects gave their written informed consent prior to study participation. This study is registered as clinical trial in ClinicalTrials.gov (NCT06781866).

### 2.3. Procedure

All studies were conducted in the subjects’ own homes. The massage sessions took place in the participants’ bedrooms prior to bedtime using a portable massage bed.

The initial screening required that all participants should score ≥16 on the Athens Insomnia Scale (AIS-8). A daily sleep questionnaire was provided for the participants to complete for the next five consecutive days prior to the initiation of this study and repeat after Sessions 2 and 3. Additionally, prior to the initiation of this study, all participants filled out a series of validated questionnaires regarding quality of life, depression scores, sleep quality, stress, and general fatigue.

During the in-house visits, the researchers arrived one hour before each subject’s usual bedtime and prepared them for this study. After each visit, the subjects were connected to the portable polysomnography system and left alone to follow their bedtime routines. The study recording was completed when the participants woke up and disconnected the system.

The CON condition was always conducted first in the order. Then, randomization ensured that each participant had an equal opportunity to be assigned first to either the REL or PLA conditions, thereby minimizing bias and preserving the integrity of the study results.

### 2.4. Questionnaires

The following questionnaires were administered:

The Athens Insomnia Scale [28], the Daily Sleep Questionnaire–5 days (DSQ-5) [29], and the Pittsburgh Sleep Quality Index (PSQI) [30] were used to assess sleep quality and sleep abnormalities [31].

The 36-Item Short-Form Survey (SF-36) quality of life questionnaire was used to assess health-related quality of life [32].

The Beck Depression Inventory (BDI) questionnaire was used to assess symptoms of depression [33].

The State–Trait Anxiety Inventory (STAI) was used to assess stress levels [34].

### 2.5. Sleep Quality and Quantity Assessment

A portable sleep-monitoring system was used to assess sleep quality and quantity (Home Sleep Test, SOMNOmedics, GmbH, Randersacker, Germany). The system recorded EEG, EOG, and EMG signals overnight. The EEG data were analyzed in 30 s epochs using the SOMNOMedics PSG analysis software (Domino panel ver. 3.0.0.8) with manual editing. The HomeSleepTest REM+ device was used for the PSG analysis, with electrodes placed according to the 10/20 system on key brain regions: Fp1 (left) and M1 for EEG and near the eyes for EOG. This setup ensured the accurate monitoring of sleep architecture and high-fidelity recordings of brain activity and eye movements.

The analysis of the sleep study was reported as follows: total sleep time (the total amount of sleep time scored during the total recording time); sleep efficiency (total sleep time/time in bed); sustained sleep efficiency (total sleep time/time in bed − sleep latency stage 2); sleep latency (the period of time it took for a person to fall asleep after they had gone to bed and tried to initiate sleep); sleep latency N1 (the period of time between wakefulness and when sleep began); sleep latency N2 (the period of time between time in bed and sleep onset stage 2); and REM latency (the amount of time elapsed between the onset of sleep and the first REM stage).

### 2.6. Massage Session

#### 2.6.1. Relaxation Massage

A relaxation type of massage was used as the main massage intervention in this study. This technique is designed to promote deep relaxation, reduce stress, and enhance overall well-being. It is characterized by the use of five primary techniques performed on the full body, including the palms and feet. Specifically, these techniques consist of effleurage (long, gliding strokes), petrissage (kneading and squeezing), tapotement (rhythmic tapping), friction (deep, circular rubbing), and vibration or shaking movements (Table 1). In addition to these, the technique also incorporates rhythmic movements and deep, light pressure to enhance relaxation and alleviate tension. It is designed to promote relaxation, increase blood circulation, relieve muscle tension, improve flexibility, promote relaxation, and reduce pain [35]. The total duration of the relaxation massage was 45 min.

#### 2.6.2. Placebo “Sham” Massage (PLA)

The sham massage (PLA) was used as a control session, without applying the deeper techniques that characterize the relaxation massage, in order to eliminate the influence of the human touch sensation on the participant’s skin (Table 2). This massage consisted only of superficial oil application movements over the entire body, without any added pressure or deep muscle manipulation. Oil was used to facilitate smooth gliding, mimicking the sensation of a full massage, but without the intensity or techniques aimed at muscle tissue relaxation. The total duration of the sham massage was also 45 min.

#### 2.6.3. Massage Oil

The massage oil was a commercially available baby oil, composed of 99% mineral oils and <1% fragrance (benzyl acetate, bicyclononalactone, isopropyl myristate, and Mefrosol). Baby oil, typically made from mineral oil (a refined petroleum product) and sometimes scented with fragrances, is generally free from naturally occurring terpenes.

### 2.7. Statistical Analysis

The statistical analysis was performed using IBM SPSS Statistics version 29.0 (IBM Corporation, Armonk, NY, USA). An independent samples *t*-test was conducted to assess significant differences in basic characteristics between male and female participants. A general linear model repeated measures test was used to examine if there were significant differences in the sleep parameters and sleep diary data between the 3 sessions. To assess normality, the Shapiro–Wilk test was used alongside graphical representations, including a normal Q-Q plot, a detrended normal Q-Q plot, and a box plot. The significance level was set at 5%. Beyond significance testing (*p*-value), the effect size was also considered to evaluate the magnitude of the effect.

### 2.8. Power Analysis

Sample size calculations were conducted using G*Power 3.1. [36]. The post hoc “GLM: repeated measures, within factors” method was used for the power analysis. Based on an expected medium effect size of 0.25 (Cohen’s f), a significance level of α = 0.05, and a desired power (1-β) of 0.82 (82%), the resulting minimum required sample size was calculated to be 20 participants. The critical F-value was 3.16 with (Ndf) = 2 and (Ddf) = 54.

## 3. Results

The subject characteristics and questionnaire scores are presented in Table 3. No statistically significant differences were observed between male and female participants (*p* > 0.05), except for in body weight (*t*(18) = 4.478; *p* < 0.001) and body height (*t*(18) = 6.478; *p* < 0.001).

The sleep parameters are presented in Table 4. A statistically significant effect was observed on sleep efficiency [F(2, 28) = 4.287; *p* = 0.034] and sustained sleep efficiency [F(2, 28) = 3.897; *p* = 0.045] across the three sessions, indicating a statistically significant effect of the relaxation massage session (*p* = 0.037 and *p* = 0.052, respectively).

**Figure 3:**
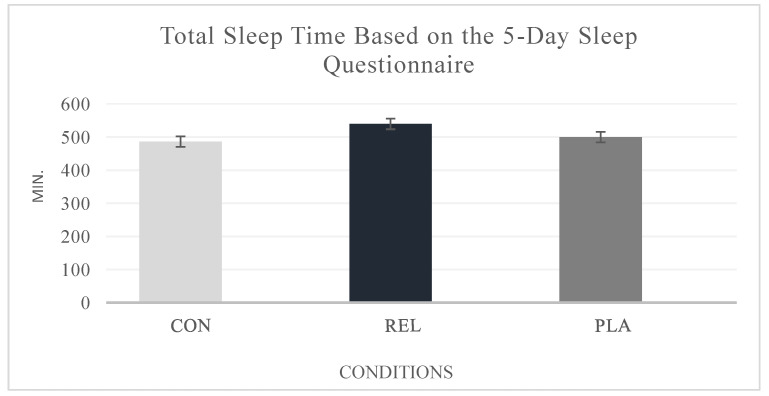
Effects of three different conditions on total sleep time based on the 5-day sleep diary. Note: No statistical differences were found. (CON): control protocol; (REL): relaxation massage protocol; PLA: “sham” massage protocol.

Additionally, the 5-Day Sleep Questionnaire (sleep diary) analysis revealed a statistically significant difference in total sleep time across the three sessions (CON, REL, and PLA) (F(2, 32) = 8.083; *p* = 0.002), with the REL condition being the most effective. Specifically, participants in the CON condition recorded a total of 486.2 min ± 1.26 of sleep over the 5-day period, followed by participants in the REL condition, who exhibited the highest total sleep time, with 539.75 min ± 1.41. Participants in the PLA condition had a total of 499.8 min ± 1.45 of sleep over the same period (Figure 3).

## 4. Discussion

To the best of our knowledge, this is the first study to evaluate the acute effects of relaxation massage (REL) prior to bedtime on sleep quality and quantity in individuals with symptoms of insomnia, using objective full-night measurements. This study showed that relaxation massage significantly improved sleep quality by improving sleep efficiency by 9.19 min (10.80%) and sustained sleep efficiency by 5.99 min (6.96%) (Table 4).

Relaxation massage, a gentler form of Swedish massage, uses light pressure through slow, rhythmic movements, incorporating techniques such as effleurage, petrissage, tapotement, friction, and vibration. These methods aim to alleviate fatigue and enhance energy levels by promoting relaxation, relieving muscular tension, and improving overall well-being [20]. One potential mechanism underlying the efficacy of relaxation massage involves the application of techniques such as myofascial release, which not only alleviates muscle tension but also enhances blood circulation. Additionally, the rhythmic and gentle pressure applied during the massage can stimulate the parasympathetic nervous system, resulting in reductions in heart rate and blood pressure [37]. Further research highlights the evolutionary significance of tactile perception throughout life, with social touch playing a critical role in emotional regulation and stress relief. Social touch, including friendly skin-to-skin contact, not only promotes relaxation but also enhances mental health [38]. This effect is particularly evident during periods of physical isolation, such as the COVID-19 pandemic, where the absence of social touch exacerbated psychological challenges. Studies like those by Dione et al. and Elias and Abdus-Saboor deepen our understanding of the biological foundation of touch and its therapeutic applications, showing that tactile stimulation contributes to reducing anxiety and sleep disorders while improving overall well-being [39,40]. Additionally, tactile interactions seem to strengthen the sense of social connection and support, emphasizing their importance for psychological well-being and coping with everyday challenges [41,42,43]. Such physiological responses may further facilitate a sense of calmness and overall well-being and create a feeling of readiness to sleep [26].

Furthermore, our study differed significantly from similar studies, such as those by V. W. H. Wong and Dedhia et al., which focused on populations with underlying medical conditions, such as heart failure and chronic kidney diseases. While these studies evaluated the effectiveness of repeated interventions, our study examined the immediate acute effects of a single relaxation massage session in individuals with severe symptoms of insomnia. Our approach is pioneering, as we objectively recorded the immediate effects of massage on sleep using a portable polysomnography system, providing precise data on brain activity during sleep. In contrast, previous studies primarily relied on subjective tools, such as the Pittsburgh Sleep Quality Index (PSQI), which lacks the reliability offered by brain activity measurements.

Polysomnographic EEG analysis revealed a positive effect of REL massage on sleep efficiency, indicating that participants fell asleep easier and faster, alleviating one of the most prominent symptoms of insomnia, which is the difficulty of falling asleep [44].

Another positive outcome of our study was the improvement of sustained sleep efficiency following the REL intervention. Sustained sleep efficiency, defined as the ratio of total sleep time to the total time spent in bed, reflects the ability to maintain uninterrupted sleep throughout the night. This positive change enhances the overall body’s recovery processes, as the time spent in bed is utilized more effectively, resulting in higher-quality sleep [45] and facilitating the physiological and psychological restorative functions essential for optimal health [46].

This improvement could translate into further reduced nocturnal awakenings and promote overall restorative sleep, something that is supported by our findings from the polysomnography study and the 5-day sleep diary [47]. Indeed, total sleep time was numerically improved by both objective and subjective measurements, indicating that REL massage could help individuals alleviate insomnia symptoms.

A number of studies have shown that massage therapy, in various settings, can significantly improve sleep quality, particularly in individuals experiencing sleep disturbances due to medical conditions; however, only one study so far has used the gold-standard methodology, which is full-night polysomnography. For instance, Kaewcum et al. examined a Swedish massage technique, indicating that it promotes relaxation and regulates brain activities related to sensory processing [48].

Moreover, studies involving cancer survivors have demonstrated that massage therapy leads to longer sleep duration and better sleep quality, as measured by both self-reported questionnaires and objective assessments [49,50]. Similarly, among nurses working rotating night shifts, massage has been shown to reduce sleep disturbances and alleviate fatigue [51]. In patients in intensive care units (ICUs), back massages have been found to improve both subjective and objective sleep quality, increasing sleep duration and reducing anxiety [52]. These findings suggest that even a brief intervention, such as a 10 min back massage, can have significant effects on sleep quality and comfort. Contrary to adults, a study on children with autism did not find any immediate improvement in sleep patterns after aromatherapy massage [53], which may be attributed to factors such as the treatment duration or the environment in which it was administered.

Additionally, studies on cardiac surgery patients have explored the effects of massage therapies on sleep and related outcomes. For instance, a trial involving foot reflexology in patients undergoing coronary artery bypass graft (CABG) surgery found no significant improvement in sleep quality, though pain intensity was significantly reduced in the intervention group [54]. Meanwhile, foot reflexology was shown to significantly improve sleep conditions in post-cardiac surgery patients, with notable improvements in sleep scores following the intervention, suggesting that these types of massage therapies can be an effective complementary approach to traditional medical care in improving sleep after surgery [55]. Additionally, massage therapy has been found to improve sleep in breast cancer patients, with significant reductions in sleep disturbances and increased restfulness at night [49]. Although individual responses may vary depending on the method of application and personal preferences, massage therapy remains a promising non-pharmacological complementary approach for improving sleep quality, particularly in those with chronic conditions or undergoing medical treatments.

Furthermore, adequate sleep ensures hormonal regulation processes that contribute to overall health and well-being [56,57]. For instance, growth hormone [58], predominantly released during deep sleep, plays a crucial role in tissue repair, muscle growth, and metabolic regulation [59]. Additionally, melatonin, known as the sleep hormone, helps regulate the circadian rhythm [60], enhancing sleep quality and facilitating the transition between sleep and wakefulness [61]. Adequate sleep also ensures the regulation of cortisol levels, the hormone associated with stress, which can reduce anxiety and depressive symptoms when maintained at optimal levels [62]. Furthermore, improved hormonal balance resulting from sufficient sleep is associated with enhanced physical health, increased energy levels, better cognitive function, and overall quality of life. Thus, sufficient sleep is essential for maintaining hormonal equilibrium and promoting a healthy state in individuals [63].

Overall, enhanced sleep quality contributes to improved physical health and hormonal regulation, including anti-inflammatory effects and psychological well-being, thereby reducing symptoms of depression and anxiety [64,65,66,67].

In the present study, we must acknowledge some significant strengths and unforeseen limitations. One of the primary strengths of our study was the analysis of the objective methodology used to assess sleep quality and quantity. Furthermore, all measurements were conducted in the participants’ homes, allowing them to feel more comfortable during the assessment. This setting ensured that the sleep data collected accurately reflected their nightly sleep patterns. In addition, the massage sessions took place prior to bedtime in the participants’ “safe and comfortable” environment, simulating even more pragmatic conditions. Finally, the blinding process that was applied when analyzing the data was very important for preventing any potential bias. Finally, the use of a PSG EEG monitoring system provided an objective measurement of brain activity that was valuable for understanding the immediate effects of massage on the brain. One limitation that requires acknowledgment was the disparity in the sample size between male and female subjects. This imbalance could have resulted in minor errors in the measurement data or inconsistencies in our results. Another limitation of our study was that the sleep assessment was not conducted by a full-polysomnography system but used a portable EEG home-based sleep-screening system. Even though this system is validated over full-night PSG, the data should still be viewed with caution. Finally, this study did not control for the menstrual cycles of the female participants.

## 5. Conclusions

In conclusion, the findings of the current study support the notion that relaxation massage prior to bedtime could be used as an effective and safe non-pharmacological complementary approach for improving sleep efficiency [68] and potentially restoring the fragmented sleep of individuals with symptoms of insomnia [69]. Consequently, these results highlight the potential of relaxation massage as a scientifically supported intervention for improving sleep patterns and, potentially, overall well-being, warranting further investigation into its broader therapeutic applications [70].

Future research should explore the long-term effects of repeated massage sessions on various populations, with a particular focus on individuals with sleep disorders and stress-related issues.

## Figures and Tables

**Table 1:** Relaxation massage protocol (full body).

Massage Technique	Rate	Time (Duration) in Minutes
Effleurage (start)	Slow to moderate (smooth and continuous strokes)	7
2. Petrissage	Moderate (kneading pressure, rhythmic)	10
3. Tapotement	Moderate to fast (quick, light tapping movements)	4
4. Friction	Moderate to deep (slow, circular movements with pressure)	6
5. Vibration	Moderate to fast (gentle or firm shaking/vibrating)	4
6. Effleurage (finish)	Slow (light strokes)	7

**Table 2:** “Sham” massage protocol (full body).

Massage Technique	Rate	Time (Duration) in Minutes
Effleurage (start)	Very slow to slow (light, smooth strokes)	10
2. Superficial gliding strokes	Slow (gentle, superficial movements)	20
3. Effleurage (finish)	Very slow (light strokes)	15

**Table 3:** Baseline characteristics.

Variable	All Participants (Mean ± SD)	Female (Mean ± SD)	Male (Mean ± SD)	*p*-Value * (Cohen’s, d)
N	20	6	14	-
Age (years)	25.5 ± 12.00	27.33 ± 7.57	30.42 ± 13.62	0.611 (0.28)
Body weight (kg)	76.1 ± 14.89	60.08 ± 10.07	83.08 ± 10.69	<0.001 (2.19)
Body height (cm)	176 ± 0.09	164 ± 0.05	181 ± 0.05	<0.001 (3.20)
BMI	24.5 ± 1.74	24.60 ± 2.62	25.51 ± 2.80	0.951 (0.33)
Athens Insomnia Scale	16.95 ± 1.08	18.16 ± 3.06	16.42 ± 1.08	0.071 (0.94)
36-Item Short-Form Health Survey (SF-36) score	85.24 ± 6.59	82.13 ± 7.86	88.34 ± 3.72	0.204 (1.00)
Beck Depression Inventory (BDI)	15.12 ± 4.38	14.75 ± 3.77	15.50 ± 5.50	0.830 (0.15)
Pittsburgh Sleep Quality Index (PSQI)	19.12 ± 5.38	18.50 ± 3.10	19.75 ± 7.54	0.770 (0.21)
Anxiety Perception Scale	21.50 ± 4.40	20.75 ± 3.86	22.25 ± 5.37	0.666 (0.32)

* Independent sample *t*-test; Note: Data are presented as means ± SD. Significance level: *p* ≤ 0.05.

**Table 4:** Sleep parameters after the 3 different sessions.

Variable				
N = 20				
	(CON) (95% CI)	(PLA) (95% CI)	(REL) (95% CI)	*p*-Value (Effect Size: Partial η^2^)
Total sleep time (TST) (hour.min.)	5.66 ± 2.0 (4.65 to 6.67)	5.82 ± 1.77 (4.92 to 6.72)	6.31 ± 1.53 (5.54 to 7.08)	0.259 (0.092)
Sleep efficiency [%]	84.54 ± 12.29 (78.3 to 90.8)	81.10 ± 12.20 (74.9 to 87.3)	89.87 ± 9.44 * (85.1 to 94.7)	0.034 (0.234)
Sustained sleep efficiency [%]	84.19 ± 13.34 (77.4 to 90.9)	84.24 ± 11.55 (78.4 to 90.1)	90.11 ± 9.4 ** (85.3 to 94.9)	0.045 *** (0.218)
Sleep latency [min.]	1.62 ± 2.33 (0.6 to 2.64)	0.65 ± 0.72 (0.334 to 0.966)	2.15 ± 3.35 (0.68 to 3.62)	0.188 (0.123)
Sleep latency N1 [min.]	1.36 ± 1.94 (0.378 to 2.34)	1.24 ±1.51 (0.476 to 2)	3.48 ± 3.97 (1.47 to 5.49)	0.057 (0.235)
Sleep latency N2 [min.]	9.82 ± 11.61 (3.94 to 15.7)	8.64 ± 11.49 (2.83 to 14.4)	14.24 ± 17.99 (5.14 to 23.3)	0.317 (0.084)
Deep sleep latency [min.]	33.08 ± 20.05 (23 to 43.2)	35.22 ± 32.41 (18.8 to 51.6)	41.52 ± 37.51 (22.5 to 60.5)	0.607 (0.037)
REM latency [min.]	98.67 ± 88.21 (54.1 to 143)	91.56 ± 81.97 (50.1 to 133)	128.70 ± 83.26 (86.6 to 171)	0.382 (0.083)
Wake index	20.26 ± 11.44 (14.5 to 26)	19.13 ± 13.86 (12.1 to 26.1)	15.80 ± 10.55 (10.5 to 21.1)	0.304 (0.081)
Wake >3 min. index	2.40 ± 1.72 (1.53 to 3.27)	2.46 ± 1.84 (1.53 to 3.39)	2.20 ± 2.54 (0.91 to 3.49)	0.858 (0.010)

Note: Data are presented as means ± SD. Significance level: *p* ≤ 0.05; CON: control; PLA: placebo, sham massage; REL: relaxation massage. Differences between the 3 protocols were assessed using a GLM repeated measures test. * Sleep efficiency: PLA vs. REL (*p* = 0.037); ** sustained sleep efficiency: PLA vs. REL (*p* = 0.045) (Bonferroni’s post hoc test); *** a trend in sustained sleep efficiency: CON vs. REL (*p* = 0.052) (Bonferroni’s post hoc test).

## Data Availability

The data presented in this study are available upon request from the corresponding author due to the fact that they contain sensitive information regarding the health history of the participants.
